# Dialysis Disequilibrium: Is Acidosis More Important than Urea?

**DOI:** 10.1155/2022/4964033

**Published:** 2022-02-22

**Authors:** Akshay Athavale, Kate R Wyburn, Paul L Snelling, Steven J Chadban

**Affiliations:** ^1^Department of Renal Medicine, Royal Prince Alfred Hospital, Sydney, New South Wales, Australia; ^2^Central Clinical School, Faculty of Medicine and Health, University of Sydney, Sydney, New South Wales, Australia

## Abstract

Dialysis disequilibrium syndrome is a severe complication associated with dialysis treatment. Manifestations may range from mild such as headache to severe such as seizures and coma. Risk factors for development include initial dialysis treatment, uraemia, metabolic acidosis, and extremes of age. We report a case of dialysis disequilibrium in a patient with a failing kidney transplant secondary to the recurrence of IgA nephropathy. Disturbance in cognition and neurologic functioning occurred six hours after the completion of initiation of intermittent haemodialysis. During two sessions of intermittent haemodialysis of 3 and 4 hours, urea was reduced by 21.9 and 17.2 mmol/L and measured serum osmolality was reduced by 25 and 14 mOsm/kg, respectively. Subsequent admission to the intensive care unit and initiation of continuous renal replacement therapy for 48 hours resulted in complete resolution of symptoms. In this case report, we discuss atypical clinical and radiologic features of dialysis disequilibrium occurring with modest reductions in urea and serum osmolality.

## 1. Introduction

Severe dialysis disequilibrium syndrome (DDS) is an increasingly rare complication following the initiation of dialysis treatment [1]. The clinical syndrome is defined by neurological symptoms occurring in close association with haemodialysis and can range from very mild, such that they go unnoticed, to severe (shown in Table 1) [2, 3]. There are numerous risk factors associated with the development of DDS (shown in Table 1) which most commonly present during or immediately following dialysis in treatment naïve patients [4]. Dialysis disequilibrium syndrome is a diagnosis of exclusion and is difficult to make due to the nonspecific nature of signs and symptoms, all of which may be present in alternative diagnoses such as encephalopathy (shown in Table 1) [3, 4].

There are three hypotheses considered to underpin the pathophysiology of DDS, and they likely do not exist in isolation. The “reverse osmosis effect” suggests that rapid removal of urea during haemodialysis establishes an osmotic gradient favouring movement of water into cells, resulting in cerebral oedema [5]. Secondly, “paradoxical brain acidosis” suggests that rapid correction of pH during haemodialysis results in a rise in the partial pressure of CO_2_ which forms carbonic acid in the brain and cerebrospinal fluid. The resulting reduction in pH displaces bound sodium and potassium, which increases intracellular osmolality and promotes cerebral oedema [1]. Finally, unidentified solutes generated in the cerebral cortex known as “idiogenic osmoles” may preserve intracerebral osmolality when haemodialysis and urea clearance are instituted, resulting in cerebral oedema [1, 6].

## 2. Case Report

A 35-year-old man presented with a 2-week history of progressively worsening fatigue, anorexia, vomiting and diarrhoea, exertional dyspnoea, and worsening oedema. Initial physical examination revealed a blood pressure of 144/94 mmHg and gross peripheral oedema of the upper and lower limbs and sacrum. Pulmonary auscultation demonstrated no features of pulmonary oedema. Background medical history was significant for living donor kidney transplantation 15 years earlier, with recurrence of IgA nephropathy and consequent nephrotic syndrome. Two months prior to the hospital admission, biochemistry demonstrated a serum creatinine (sCr) of 572 umol/L, urea of 30 mmol/L, potassium of 4.7 mmol/L, bicarbonate of 16 mmol/L, corrected calcium of 1.96 mmol/L, and phosphate of 2.82 mmol/L.

Following admission to the hospital, acute on chronic kidney injury was noted with an sCr of 1439 umol/L and urea of 53.2 mmol/L. Serum potassium was raised at 6.1 mmol/L and bicarbonate was critically low at 6 mmol/L with a pH of 7.06. Corrected calcium had reduced further to 1.67 mmol/L, and phosphate had increased to 5.97 mmol/L. Serum sodium and blood glucose were normal, and measured serum osmolality was 337 mOsm/kg. A complete blood count demonstrated anaemia with a haemoglobin of 98 g/L.

Initial medical management was instituted over the first 36 hours and involved parenteral loop diuretic therapy for fluid overload, oral bicarbonate replacement for acidosis, and both insulin dextrose treatment and oral potassium exchange resins for hyperkalaemia. Oral calcitriol and calcium carbonate were administered for hypocalcaemia. Despite medical management, bicarbonate remained unchanged at 6 mmol/L with a pH of 7.13, and phosphate worsened to 6.03 mmol/L. Serum potassium and corrected calcium both improved to 5.1 mmol/L and 1.72 mmol/L, respectively. Measured serum osmolality increased to 341 mOsm/kg. The patient deteriorated further, developing uraemic encephalopathy as evidenced by delirium, fluctuating levels of consciousness, dysarthria, and asterixis. A widespread tremor was also present. The patient was urgently initiated on haemodialysis treatment.

The first haemodialysis treatment was 3 hours in duration, with an ultrafiltration volume of 3000 mL. The dialysis membrane used was Polyflux 140H, and the dialysate was Baxter CX265G. The dialysate flow rate was 500 mL/min and the blood flow rate was 200 mL/min. The patient demonstrated a significant improvement in symptoms within 24 hours following dialysis, with complete resolution of cognitive and speech deficits but persisting tremor. Twenty-four hours later, he underwent a second session of haemodialysis of 4 hours' duration with an ultrafiltration volume of 4000 mL. The dialysis membrane used was Polyflux 140H, and the dialysate was Baxter CX275G. The dialysate flow rate was 500 mL/min and the blood flow rate was 250 mL/min. Immediately following the second session of dialysis, complete resolution of all symptoms was achieved, and the patient was planned for discharge home. Dialysis prescription and biochemistry before and after both dialysis sessions are summarised in Table 2.

Six hours after completing the second session of haemodialysis, the patient developed severe hypertension with a blood pressure of 187/102 mmHg (143/92 mmHg following dialysis) and was found to be stuporous with hyperreflexia and sustained clonus. There was no response to visual threat bilaterally; however, he was able to follow commands. As a result of this marked deterioration, he was intubated and transferred to the intensive care unit (ICU) for further evaluation.

**Figure 1:**
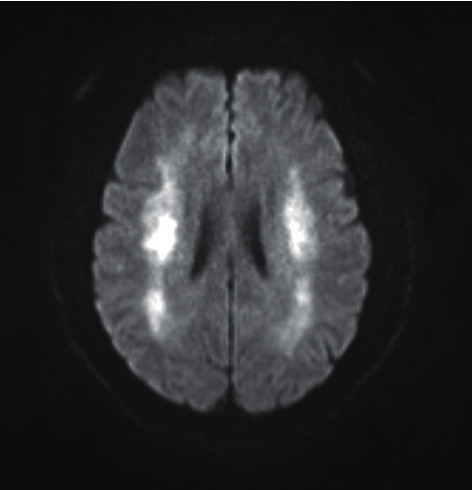
Magnetic resonance imaging findings demonstrating symmetrical bilateral supratentorial restriction in keeping with leukoencephalopathy.

Contrast computed tomography of the brain showed no abnormality, and magnetic resonance imaging of the brain revealed bilateral symmetric diffusion restriction in the supratentorial white matter of both cerebral hemispheres without evidence of overt cerebral oedema or features of posterior reversible encephalopathy syndrome (PRES; shown in Figure 1). Lumbar puncture demonstrated no evidence of meningoencephalitis or autoimmune encephalitis, and an electroencephalogram (EEG) had no features of seizure activity. Ammonia concentrations were not elevated and cryptococcal testing was negative.

The patient was diagnosed with dialysis disequilibrium syndrome and commenced on continuous venovenous haemodiafiltration (CVVHDF) to manage severe fluid overload and hypertension, which was continued for a total of 48 hours. During this time, urea was slowly reduced from 27.8 mmol/L to 11 mmol/L, bicarbonate was increased from 18 mmol/L to 28 mmol/L, and corrected calcium increased from 2.11 mmol/L to 2.54 mmol/L. The ultrafiltration volume achieved was 6300 mL, and he was subsequently extubated and demonstrated complete resolution of all signs and symptoms. Forty-eight hours after leaving the ICU, he was discharged home on maintenance intermittent haemodialysis.

Written informed consent was obtained from the patient for the publication of this case report and any accompanying images.

## 3. Discussion

We report a case of dialysis disequilibrium syndrome manifesting six hours after the completion of the second haemodialysis session in a patient with a failing kidney transplant. The dialysis disequilibrium was the most likely diagnosis as other differentials including meningoencephalitis, autoimmune encephalitis, PRES, and seizure disorder were excluded. Most commonly, dialysis disequilibrium occurs during or immediately after haemodialysis, and late presentations such as in our case are less common [1, 7].

Dialysis disequilibrium syndrome is a rare and potentially fatal complication of dialysis treatment [8, 9]. Signs and symptoms range from mild such as nausea and vomiting to severe and life-threatening such as seizures and coma [1]. While DDS is most commonly associated with first haemodialysis treatment, there are reports of DDS in patients treated with continuous renal replacement therapies (RRTs) such as continuous venovenous haemodialysis (CVVHD) and continuous venovenous haemofiltration (CVVH) [2].

Development of DDS is variable and dependent on both the extent and rate of change of osmotically active solutes such as urea [2]. Central to the underlying pathogenesis of DDS is the establishment of an osmotic gradient between the blood compartment and the brain and cerebrospinal fluid (CSF) [3]. In states of chronic uraemia, a steady state is established between compartments, preventing significant fluid shifts. Dialysis treatment, both intermittent and continuous, will remove urea from the central blood compartment, resulting in a net movement of fluid into the brain, causing cerebral oedema [10, 11]. Rodent models have demonstrated an upregulation of aquaporin channels and downregulation of rapid urea transporters in the brain as a result of uraemia, and these phenomena may also contribute to the development of cerebral oedema seen in DDS [12].

The “paradoxical brain acidosis” hypothesis suggests that the rapid correction of pH by dialysis results in a rise in the partial pressure of carbon dioxide which forms carbonic acid in the brain. The reduced intracerebral pH causes displacement of bound cations such as sodium and potassium, thereby causing a rise in intracerebral osmolality. This situation favours the movement of water into the brain, further compounding cerebral oedema [13]. Finally, “idiogenic osmoles” which are intracellular osmoles generated as a compensatory mechanism to maintain intracerebral osmolality in states of hyperosmolality (e.g., hyperglycaemia and uraemia) may drive movement of water into the brain during dialysis [14].

Autopsy and neuroimaging data in both animals and humans have demonstrated evidence of cerebral oedema in DDS [15, 16]. Importantly, however, while cerebral oedema is frequently seen in DDS, other patterns of injury including demyelination and leukoencephalopathy have been demonstrated [17, 18]. Leukoencephalopathy has many causes including cerebral oedema, immunosuppressive medications, central nervous system infections, and impaired cellular immunity [17]. In our case, imaging findings did not demonstrate overt cerebral oedema, but were suggestive of an acute leukoencephalopathy, which was likely a consequence of diffuse interstitial cerebral oedema that may be seen in DDS [18].

There is a lack of data demonstrating the optimal parameters to reduce the risk of DDS. It is generally accepted that reducing urea by approximately 40% over a 2-hour haemodialysis session is reasonable, though this is not evidence-based [9]. In our case, urea was reduced by 39% over the initial 3-hour haemodialysis session and by 41% over the following 4-hour session. Further, ensuring that the reduction in plasma osmolality does not significantly exceed 24 mOsm/kg may be of benefit in preventing DDS [3]. In our case, serum osmolality was reduced by 25 mOsm/kg and 14 mOsm/kg after the first and second haemodialysis sessions, respectively. This suggests that perhaps in our case, rapid correction of metabolic acidosis rather than urea reduction may have contributed more significantly to the development of DDS.

While DDS has been reported to occur up to 24 hours following dialysis, most commonly, it presents towards the end or immediately after completing dialysis. In our case, signs and symptoms manifested 6 hours after completion of dialysis. Given that the rate of reduction of urea was in line with accepted recommendations, it is unclear as to why signs and symptoms were delayed. It is likely that development of DDS in our case was more in keeping with the “paradoxical brain hypothesis” as bicarbonate increased by 14 mmol/L over 7 hours of dialysis. Therefore, as blood bicarbonate increased, carbon dioxide diffused slowly into the brain, reducing pH and slowly displacing tissue-bound cations. This would likely be a slower process than simply establishing an osmotic gradient secondary to urea removal and may explain the delayed onset of signs and symptoms.

Treatment of DDS is supportive and may involve discontinuation of dialysis [3]. In cases such as ours, where neither urea nor osmolality were reduced at rates significantly greater than recommended [9], consideration should be given to the use of a lower bicarbonate dialysate, to limit the rate of correction of metabolic acidosis. Dialysate solutions vary globally, but solutions containing less than 30 mmol/L of bicarbonate are available [19] and may represent a safer choice in cases such as ours. Continuous RRT may also represent a reasonable approach, but DDS occurring with this treatment has also been reported [2]. Furthermore, in patients at high risk of developing DDS, shorter dialysis sessions of 2 hours with slower blood flow rates of 150–250 mL/min, a smaller dialyzer, and both a lower target for urea reduction and a slower rate of urea reduction during the initial dialysis may be appropriate [9, 20].

Additionally, in patients considered high risk of DDS, such as those with risk factors shown in Table 1, antiepileptic drugs (AED) have been used as both a preventative and a therapeutic treatment [21]. Currently, there are no randomised data to support the use of prophylactic AEDs in DDS, and their use does little to address the underlying pathophysiology of cerebral oedema, which is central to DDS [20, 21]. Data from neurological literature suggest that newer AEDs, such as levetiracetam, may be preferred due to fewer drug interactions, but the dose and duration of treatment remain unclear [22]. Acute seizures occurring as a consequence of DDS may be terminated using benzodiazepines or other AEDs [21].

Finally, the use of exogenous solutes such as intravenous mannitol (1 mg/kg), higher dialysate urea, or glucose can be administered to counteract the reduction in urea and serum osmolality associated with dialysis [21]. While there are no randomised data on this approach, current evidence suggests that it may reduce the incidence of symptoms of DDS [4] and could be considered in patients at high risk of DDS.

## 4. Conclusion

We have presented a unique case of dialysis disequilibrium syndrome where the classic precipitant of extensive urea reduction on dialysis was absent. We propose that the 6-hour delay to the onset of signs and symptoms was due to the correction of metabolic acidosis, in keeping with the “paradoxical brain acidosis” hypothesis. Dialysis disequilibrium is an increasingly rare complication of treatment, but awareness of risk factors, timing, spectrum of severity, and treatment are important to all physicians prescribing dialysis.

## Figures and Tables

**Table 1:** Signs, symptoms, risk factors, and differential diagnoses for dialysis disequilibrium syndrome.

Signs	Symptoms	Risk factors	Differential diagnoses
Asterixis	Headache	New dialysis initiation	Malignant hypertension
Altered mental status	Nausea	Chronic kidney disease	Posterior reversible encephalopathy syndrome (PRES)
Seizures	Vomiting	Urea >60 mmol/L	Cerebrovascular accident
Coma	Muscle cramps	Metabolic acidosis	Hyponatraemia
	Confusion	Extremes of age	Uraemia
	Tremor	Preexisting neurologic disorder	Intracranial haemorrhage
	Visual disturbance	Conditions associated with cerebral oedema	Hypoglycaemia
			Meningoencephalitis
			Seizure disorder
			Uraemic encephalopathy

**Table 2:** Dialysis prescription, before and after haemodialysis biochemistry.

	Before HD (1)	After HD (1)	Before HD (2)	After HD (2)
Dialysis membrane	Polyflux 140H		Polyflux 140H	
Dialysate (mmol/L)	Baxter CX265G (K^+^ 2, Ca^2+^ 1.65, Mg^2+^ 0.5, Na^+^ 140, HCO_3_^−^ 34)		Baxter CX275G (K^+^ 2, Ca^2+^ 1.75, Mg^2+^ 0.5, Na^+^ 140, HCO_3_^−^ 34)	
DFR/BFR (mL/min)	500/200		500/250	
Creatinine (umol/L)	1535	929	1201	858
Urea (mmol/L)	55.9	34.0	41.8	24.6
Sodium (mmol/L)	139	142	140	143
Potassium (mmol/L)	5.1	3.3	3.9	3.6
Bicarbonate (mmol/L)	6	12	14	20
Corrected calcium (mmol/L)	1.72	2.11	1.82	2.20
Phosphate (mmol/L)	6.03	3.72	5.40	3.48
Osmolality (mOsm/kg)	341	316	324	310
Ultrafiltration (mL)	N/A	3000	N/A	4000

HD: haemodialysis; DFR: dialysate flow rate; BFR: blood flow rate; mmol/L: millimoles per litre; K^+^: potassium; Ca^2+^: calcium; Mg^2+^: magnesium; Na^+^: sodium; HCO_3_^−^: bicarbonate.

## Data Availability

There are no data available for this work.
